# Viral transmission risk factors in an Egyptian population with high hepatitis C prevalence

**DOI:** 10.1186/s12889-015-2369-y

**Published:** 2015-10-07

**Authors:** Mary Kate Mohlman, Doa’a A. Saleh, Sameera Ezzat, Mohamed Abdel-Hamid, Brent Korba, Kirti Shetty, Sania Amr, Christopher A. Loffredo

**Affiliations:** Lombardi Cancer Center, Georgetown University, 3970 Reservoir Rd, Washington, DC 20057 USA; Cairo University, Cairo, Egypt; Menoufiya University, Shibin El Kom, Egypt; Minia University, Minia, Egypt; University of Maryland School of Medicine, Baltimore, MD USA

**Keywords:** HCV, Egypt, Prevalence, Transmission

## Abstract

**Background:**

Egypt has the world’s highest prevalence of infection with hepatitis C virus (HCV), which is a major cause of hepatocellular carcinoma. The high HCV prevalence is largely attributed to the parenteral antischistosomal therapy (PAT) campaigns conducted from the 1950s through the 1980s; however, the primary modes of transmission in the post-PAT period are not well known. In this study we examined the associations between HCV prevalence and exposures to risk factors, including PAT, in a high HCV prevalence population.

**Methods:**

Using a cross-sectional design, we examined the associations between demographic characteristics and risk factors for HCV transmission and HCV positivity prevalence among a sample of Egyptian residents. Data were collected through an interview-administered survey, and the association estimates were determined using *χ*^2^ and logistic regression.

**Results:**

The highest HCV positivity prevalence was observed in cohorts born before 1960, and declined precipitously thereafter; whereas the proportion of subjects reporting PAT remained relatively stable. Being male, having a rural residence, and having received PAT were all associated with HCV positivity; however, PAT alone could not account for the high prevalence of HCV.

**Conclusions:**

In Egypt, PAT and other transmission factors yet to be identified, as well as cohorts born before the 1960s and infected with HCV, are most likely the main contributors to the current HCV endemic.

## Background

Hepatitis C virus (HCV), a blood-borne pathogen, is a major cause of hepatocellular carcinoma (HCC). The incidence of this cancer is increasing worldwide [1], particularly in Egypt, which has the world’s highest HCV prevalence. From 1997 to 2001, Egypt’s incidence of liver cancer doubled [2], and a recent estimate of incidence is 38.1 per 100,000 for males and 14.1 per 100,000 for females [3]. Those chronically infected with HCV are 15 to 20 times more likely to develop HCC than those who are not infected [4].

In Egypt, the prevalence of the two major biomarkers of HCV - the anti-HCV antibodies and HCV RNA seropositivity - is estimated at 14.7 and 9.8 %, respectively, in the general population; but it is much higher among those over age 50 (>35.0 % for anti-HCV antibodies and > 25 % for HCV RNA) [5]. The source of this epidemic has largely been attributed to the parenteral antischistosomal therapy (PAT) campaigns that took place from the 1950s through the 1980s [6]; however, the primary modes of transmission in the post-PAT period are not well known. Iatrogenic sources have been considered key contributors, especially among the elderly who are at risk of chronic diseases and thus potential medical treatments. While Egypt has made great strides in improving infection control, an overburdened, under-funded healthcare system does not always promote optimal measures [7]. This especially holds true where an informal healthcare system operates in parallel to the official one [8]. Breban et al., using a dynamic model of HCV transmission, postulated that ongoing HCV transmission is fueled in large part by a small group of infectious individuals with high rates of medical treatments and healthcare provision with suboptimal infection control, resulting in the lasting impact of iatrogenic transmission [9].

In this study, we examined the associations between demographic characteristics, risk factors for HCV transmission, and HCV seropositivity prevalence. Our aim was to understand how HCV infection among the oldest group in our study sample could contribute to current transmission trends.

## Methods

### Study population and data collection

For the present study we used a cross-sectional analysis to examine the control group that participated in a case–control study on HCC (hereafter referred to as the HCC study) conducted in Egypt between 1999 and 2010. Details of the HCC study enrollment, case confirmation, interview procedures, and participation have been previously described in detail [10]. Briefly, participants were residents of Egypt who lived in the Cairo-Giza metropolitan area and in surrounding governorates. Cases, defined as those diagnosed with HCC, were recruited at the National Cancer Institute of Cairo University. Controls, recruited at Cairo University’s orthopedic hospital, were selected to frequency-match the cases by gender, age category (5-year age groups), and current residence category (i.e. rural or urban). Additional rural male controls were recruited at public health clinics in villages of the Qalyubia Governorate, north of Cairo, to ensure adequate matching by residence [10].

Inclusion criteria included being over the age of 17 and having been resident in Egypt for at least a year. All participants signed informed consent forms or had a witness sign if they were illiterate. Information on those who refused to participate and their reason was also documented. The protocol was approved by the Institutional Review Boards of Georgetown University, the University of Maryland, Baltimore, and Cairo University. An interviewer administered the questionnaire face-to-face to participants and recorded their answers. Additionally, researchers collected ten milliliters of blood from participants for serological testing of HCV antibodies and HCV RNA, as well as hepatitis B virus (HBV) core antibodies and HBV surface antigen, as previously described [10].

Between 2001 and through 2010, we recruited 1764 controls for the HCC study. We collected data on demographics and potential environmental risk factors; i.e., tobacco smoking, alcohol drinking, environmental tobacco smoke, pesticides, and water and air pollution [10]. In addition, the HCC study inquired about five risk factors with the potential for exposure to HCV-contaminated blood or instruments: receiving PAT, receiving a blood transfusion, donating blood, receiving therapeutic injections, and a diagnosis of diabetes, which often leads to insulin injections. This study focused on the demographic, PAT, and medical variables with potential for exposure to HCV.

### Assessment of HCV status

Detailed descriptions of testing have been previously described [10, 11]. Briefly, ten milliliters of blood were collected from each patient by venipuncture. Blood was separated and the serum divided into three aliquots, one of which was analyzed immediately, while the other two were stored at −80 °C. To identify the presence of HCV antibodies, we used third generation ELISA screening tests (Abbott Laboratories) [10]. A reverse transcription (RT)-PCR based method, developed by Abel-Hamid and colleagues [11], was used to test for HCV RNA. Where the ELISA test was positive and the RT-PCR test was negative, the latter test was repeated after re-extracting and purifying the RNA from the whole blood specimen [10]. HCV positivity in this study was defined as positive for HCV antibodies and/or HCV RNA.

### Statistical analysis

Data analysis included descriptive statistics of the study sample by age, gender, birthplace, current residence, education, and medical history. Pearson *χ*^2^ tests were used to compare HCV status between groups. Logistic regression was used to generate the unadjusted and adjusted odds ratio (OR and AOR) and 95 % confidence interval (CI) of the association between HCV status and each of the above variables. The two-sample proportion test was used to compare HCV status and PAT prevalence. Since the data were collected over a 10-year period, we converted age at interview to year of birth to appropriately apportion the study sample into 10-year age groups. Stata 13 was used for all statistical analysis.

## Results

### Demographic characteristics

Table 1 shows the distribution of the sample population among the age groups. The years of birth ranged from 1913 to 1992 with a median year of 1959. Overall, 62.0 % of the sample was male, and male predominance was present in all age groups. Rural residence at birth accounted for 77.3 % of the oldest group and declined steadily among the younger groups.

**Table 1:** Gender, birthplace, and current residence of the different age groups of a sample of the Egyptian population

Age groups (year of birth)	N (%)	% Male	Birthplace (% rural)	Residence at time of interview (% rural)
Overall	1764 (100)	62.0	65.2	56.5
1913–1939	176 (10.0)	69.9	77.3	56.6
1940–1949	266 (15.1)	58.8	69.9	56.4
1950–1959	449 (25.5)	60.8	68.6	59.0
1960–1969	332 (18.8)	59.9	63.3	56.3
1970–1979	255 (14.5)	67.5	57.1	52.2
1980+	286 (16.2)	58.7	57.4	56.6

Approximately 65.2 % of the participants were born in a rural area, but only 56.5 % lived in rural area at the time of the interview (Table 1), indicating urban migration. Overall, 18.2 % of respondents had not relocated from their birthplace at the time of the interview. Of these, 230 (72.1 %) lived in rural areas. Of those who had moved, 7.1 % moved from an urban to a rural area, and 17.8 % moved from a rural to an urban area.

### Risk factors for HCV transmission

Among the 1764 participants, the prevalence of anti-HCV antibodies and HCV RNA was 29.8 and 22.9 % respectively. Table 2 shows HCV positivity prevalence among the different demographic groups; the highest prevalence was observed among males, those born before 1960, those born in rural areas, those living in rural areas at the time of interview, and those with the least amount of formal education.

**Table 2:** Associations between HCV positivity (anti-HCV antibodies and/or HCV RNA positive) and different demographic characteristics among a sample of the Egyptian population

Study population characteristic	HCV Prevalence (%)	Unadjusted OR (95 % CI)	Adjusted OR (95 % CI)^a^
Overall	29.8	NA	NA
Gender			
Male	32.2	Ref	Ref
Female	25.3	**0.71 (0.56, 0.91)**	**0.65 (0.48, 0.86)**
Age group			
1913–1939	40.5	Ref.	Ref
1940–1949	45.0	1.20 (0.79, 1.82)	1.32 (0.85, 2.04)
1950–1959	42.1	1.07 (0.73, 1.57)	1.17 (0.78, 1.76)
1960–1969	27.2	**0.55 (0.36, 0.83)**	**0.59 (0.38, 0.91)**
1970–1979	15.0	**0.26 (0.16, 0.43)**	**0.27 (0.16, 0.45)**
1980+	6.8	**0.11 (0.06, 0.19)**	**0.10 (0.06, 0.20)**
Education			
No schooling, literacy only, or religious school	35.5	Ref.	Ref.
Primary through secondary	27.6	**0.69 (0.54, 0.88)**	1.21 (0.91, 1.61)
Higher education	21.8	**0.55 (0.46, 0.66)**	0.90 (0.51, 1.59)
Migration pattern			
Current same as birth – rural	35.1	Ref.	Ref.
Current same as birth - urban	15.7	**0.34 (0.18, 0.66)**	**0.41 (0.20, 0.84)**
Urban to urban migration	17.7	**0.40 (0.27, 0.58)**	**0.32 (0.21, 0.50)**
Rural to rural migration	41.2	1.30 (0.92, 1.82)	1.03 (0.70, 1.51)
Rural to urban migration	32.2	0.88 (0.59, 1.3)	**0.64 (0.41, 0.99)**
Urban to rural migration	12.3	**0.26 (0.12, 0.57)**	**0.21 (0.09, 0.48)**
Birthplace			
Urban	16.7	Ref.	Ref.
Rural	37.5	**3.00 (2.30, 3.91)**	**2.36 (1.68, 3.31)** ^b^
Residence at time of interview			
Urban	22.2	Ref.	Ref.
Rural	36.9	**2.04 (1.62, 2.57)**	**1.35 (1.01, 1.83)** ^b^

^a^All characteristics listed in the first column were included in the adjusted logistic regression model except for “Birthplace” and “Residence at time of interview”

^b^In these models adjustement was made for all variables in first column except “Migration pattern”

ORs in **bold** indicate statistical significance

**Fig. 1:**
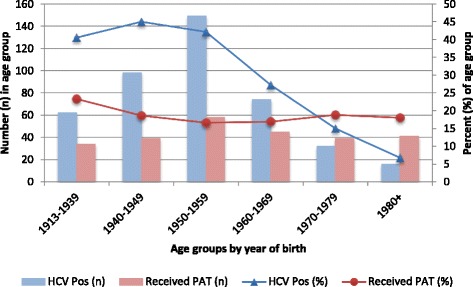
Comparison between the number and percent of subjects who received parenteral antischistosomal therapy (PAT) and the number and percent of those who were HCV positive (HCV Pos) for either HCV anti-bodies and/or RNA in each of the age group

Figure 1 provides information on the number and proportion of subjects who reported receiving PAT over time and of those who were HCV-positive among each age group. Two different trends are apparent: (1) the proportion of HCV-positive subjects exceeded 40 % in the three oldest birth groups, and declined precipitously thereafter (i.e. after 1959); whereas (2) the proportion reporting PAT remained relatively stable. HCV positivity prevalence was significantly higher than PAT prevalence for the 4 oldest age groups (*p-value < 0.001*). There was no statistically significant difference for those born between 1970 and 1979. For those born in 1980s and after, PAT prevalence was significantly higher than HCV prevalence (*p-value <0.001*).

In the regression model, we found that females were 37 % less likely than males to be HCV positive after adjustment for age, residence (both at birth and interview time), and education. We found no statistically significant difference in HCV positivity between those born before 1940 and those born between 1940 and 1959. For those born after 1959, the odds of being HCV positive decreased gradually with each birth decade (Table 2). Increasing educational attainment lost its statistical significance as an indicator of HCV positivity after adjustment for age, gender, and migration status (Table 2). We also observed that a rural birthplace was associated with being HCV positive (AOR 2.36 (95 % CI 1.68, 3.31)), as was current residence in rural areas (AOR 1.35 (95 % 1.01, 3.31)), although to a lesser extent (Table 2). When we assessed the effects of migration and its patterns, using being born and living in the same rural residence as the reference group, all groups had significantly lower odds of being HCV positive, except the group with a rural-to-rural migration pattern, for which there was no significant difference (Table 2).

The age group with the highest lifetime exposure to transmission risk factors included those born between 1950 and 1959 (22.7 % exposed to PAT; 22.4 % exposed to blood transfusions, 25 % donated blood, 34.2 % had diabetes, and 34.8 % had frequent or regular injections). In this group, the prevalence of HCV positivity was high among those who were exposed to PAT (44.8 %); received blood transfusion (43.5 %); donated blood (40.0 %); were diagnosed with diabetes (46.0); and those who received frequent or regular injections (34.4 %).

In contrast, those born after 1980 had modest to low exposure to risk factors (16 %–17 % were exposed to PAT, blood transfusion, and blood donation; 1.6 % had diabetes; and none had frequent or regular injections). Of those exposed to these risk factors, the prevalence of HCV ranged from 0.0 % (diabetes and frequent or regular injections) to 8.6 % (blood donation). PAT and blood transfusion fell in the middle with 4.9 and 6.7 % prevalence respectively.

In Table 3, logistic regression revealed that only PAT exposure was significantly associated with HCV positivity, after adjustment for age group, gender, and education. A diabetes diagnosis had a significant unadjusted OR of 1.61 (95 % CI 1.16, 2.22), but the association lost its significance after adjustment for other covariates. Additionally, of those diagnosed with diabetes, approximately half received insulin therapy, but there was no significant difference (*χ*^2^ test p-value: 0.25) in HCV positivity prevalence between those who did and did not receive insulin.

**Table 3:** Associations between HCV positivity (anti-HCV antibodies and/or HCV RNA positive) and exposures to potential routes of HCV transmission in a sample of the Egyptian population

Route of Transmission	Exposure Response	Exposure, N	HCV Prevalence, %	Unadjusted OR (95 % CI)	Adjusted OR (95 % CI)^a^
Received PAT	No	1408	23.1	Ref.	Ref.
	Yes	305	60.2	**1.62 (1.22, 2.16)**	**1.54 (1.14, 2.11)**
Blood Transfusion	No	1417	29.1	Ref.	Ref.
	Yes	324	32.1	1.16 (0.87, 1.53)	1.31 (0.96, 1.79)
Blood Donation	No	1252	31.4	Ref	Ref.
	Yes	487	25.7	**0.76 (0.59, 0.98)**	0.76 (0.57, 1.02)
Diabetes Diagnosis	No	1251	28.5	Ref.	Ref.
	Yes	184	39.1	**1.61 (1.16, 2.22**)	1.23 (0.87, 1.76)
Number of Injections	Less than 10 injections	116	24.1	Ref	Ref.
	10 or more injections	1209	30.0	1.35 (0.87, 2.10)	1.24 (0.76, 2.02)
	Has injections frequently/continuously	92	31.5	1.45 (0.78, 2.67)	1.23, (0.63, 2.41)

^a^Adjusted for sex, age group, birthplace, residence at time of interview, and education; ORs in **bold** indicate statistical significance

## Discussion

Our findings suggest that in Egypt, 1) PAT and other risk factors yet to be identified were historically and remain important for HCV transmission; and 2) cohorts born before the 1960s with high HCV prevalence are most likely the main contributors to the current HCV endemic. This last point is in line with the proposal put forward by Breban et al. that a small infectious population with high rates of medical exposures is a significant source of transmission in Egypt [9].

Our study sample characteristics are comparable to those of the nationally representative sample recruited and tested for HCV positivity through the Egypt Demographic and Health Survey (EDHS) [5] for health policy purposes. Although we found the overall prevalence of anti-HCV antibodies and HCV RNA to be much higher in our study population (29.6 and 22.9 % respectively) than in the EDHS (14.7 and 9.8 %), the prevalence was similar when we examined comparable age groups and gender. Indeed, of those born between 1950 and 1959 in our study population, the prevalence of HCV positivity was 49.5 % for males and 30.9 % for females, compared to 46.3 and 30.8 % respectively in this age group in the EDHS. Furthermore, the EDHS estimated that 53.9 % of males and 54.8 % of females lived in rural areas at the time of the study, proportions that are close to those we found of 58.9 and 52.6 %, respectively. And both our study and the EDHS found higher prevalence of HCV positivity in rural than in urban communities. The discrepancy in the overall prevalence of HCV positivity is most likely due to the EDHS sample having higher proportions of young individuals (testing for HCV was limited to those 15 to 59 years old), who tended to be more educated than the older ones and have lower HCV prevalence. Our sample population had a higher proportion of those who were living in rural areas and of those with less primary schooling. Nonetheless, both our study and the EDHS showed that less education and rural residence were associated with higher HCV positivity prevalence [12].

We found PAT to be the only risk factor that was significantly associated with HCV positivity; however in comparing the proportion of those who were HCV positive to the proportion of those who had received PAT (Fig. 1), we noted that the prevalence of HCV positive cases in each age group, except the two youngest, was much higher than the proportion of those exposed to PAT. Therefore, PAT cannot fully explain the high HCV prevalence in Egypt; transmission that occurred through additional routes, perhaps iatrogenic and community based, is highly likely [13]. Such a possibility is supported by our findings that the groups with high HCV positivity prevalence also had high prevalence of lifetime exposure to blood transfusion, blood donation, diabetes diagnosis, and frequent or regular injections. Another explanation for the trends in Fig. 1 is that PAT injection practices were riskier in the earlier half of the 20th century but became safer thereafter.

Our study did not find a statistically significant association between HCV positivity and blood transfusion, blood donation, therapeutic injections, and diabetes diagnosis (after adjustment for demographic factors), in contrast to other studies that focused on these risk factors [12, 14–16]. However, the association between aging populations and increased healthcare utilization [5], which in turn increase the risk for HCV transmission, and the high HCV positivity prevalence among individuals born before 1960 suggest that the latter cohorts may substantially contribute to the ongoing HCV transmission in Egypt; a plausibility that is consistent with Breban et al.’s concept [9].

One of the study’s limitations was that the main focus of the HCC study was on cancer risk factors; therefore it did not assess all possible HCV transmission risk factors such as the informal healthcare sector, intrafamilial transmission, and exposure risks identified by other researchers [17–20]. The lack of association between some of the known transmission risk factors and HCV positivity, not just in our study but in many others [14, 15, 21, 22], highlights the difficulty in studying an often asymptomatic disease where the time point and source of infection are difficult to identify.

## Conclusions

In summary, we found that PAT and aging cohorts of individuals infected with HCV are not the only contributing factor to the current HCV endemic in Egypt, although they play major roles; additional factors of viral transmission are yet to be identified. Therefore, research investigating the relationships of current HCV prevalence rates to historical transmission risk factors among different segments of Egypt’s population are warranted. Temporal changes in iatrogenic and community based modes of transmission may underlie current patterns of prevalence, but remain to be fully elucidated.
